# Genetic Architecture of Carcass and Meat Quality Traits in Montana Tropical^®^ Composite Beef Cattle

**DOI:** 10.3389/fgene.2020.00123

**Published:** 2020-02-27

**Authors:** Laís Grigoletto, José B. S. Ferraz, Hinayah R. Oliveira, Joanir P. Eler, Fernando O. Bussiman, Barbara C. Abreu Silva, Fernando Baldi, Luiz F. Brito

**Affiliations:** ^1^Department of Animal Sciences, College of Animal Sciences and Food Engineering, University of São Paulo, Pirassununga, Brazil; ^2^Department of Animal Sciences, Purdue University, West Lafayette, IN, United States; ^3^Department of Animal Sciences, College of Agricultural and Veterinary Sciences, São Paulo State University, Jaboticabal, Brazil

**Keywords:** candidate genes, composite cattle, crossbreeding, genomic regions, single-step Genome-Wide Association Studies (ssGWAS), Genomic Best Linear Unbiased Prediction (GBLUP), tropical beef cattle

## Abstract

The Montana Tropical^®^ Composite is a recently developed beef cattle population that is rapidly expanding in Brazil and other tropical countries. This is mainly due to its improved meat quality and adaptation to tropical climate conditions compared to Zebu and Taurine cattle breeds, respectively. This study aimed to investigate the genetic architecture of ultrasound-based carcass and meat quality traits in Montana Tropical^®^ Composite beef cattle. Therefore, we estimated variance components and genetic parameters and performed genome-wide association studies using the weighted single-step Genomic Best Linear Unbiased Prediction (GBLUP) approach. A pedigree dataset containing 28,480 animals was used, in which 1,436 were genotyped using a moderate-density Single Nucleotide Polymorphism panel (30K; 30,105 SNPs). A total of 9,358, 5,768, 7,996, and 1,972 phenotypic records for the traits *Longissimus* muscle area (LMA), backfat thickness (BFT), rump fat thickness (RFT), and for marbling score (MARB), respectively, were used for the analyses. Moderate to high heritability estimates were obtained and ranged from 0.16 ± 0.03 (RFT) to 0.33 ± 0.05 (MARB). A high genetic correlation was observed between BFT and RFT (0.97 ± 0.02), suggesting that a similar set of genes affects both traits. The most relevant genomic regions associated with LMA, BFT, RFT, and MARB were found on BTA10 (5.4–5.8 Mb), BTA27 (25.2–25.5 Mb), BTA18 (60.6–61.0 Mb), and BTA21 (14.8–15.4 Mb). Two overlapping genomic regions were identified for RFT and MARB (BTA13:47.9–48.1 Mb) and for BFT and RFT (BTA13:61.5–62.3 Mb). Candidate genes identified in this study, including *PLAG1*, *LYN, WWOX*, and *PLAGL2*, were previously reported to be associated with growth, stature, skeletal muscle growth, fat thickness, and fatty acid composition. Our results indicate that ultrasound-based carcass and meat quality traits in the Montana Tropical^®^ Composite beef cattle are heritable, and therefore, can be improved through selective breeding. In addition, various novel and already known genomic regions related to these traits were identified, which contribute to a better understanding of the underlying genetic background of LMA, BFT, RFT, and MARB in the Montana Tropical Composite population.

## Introduction

Both carcass and meat quality traits are paramount for optimizing the profitability of the beef cattle industry. These traits are influenced by diet and feeding practices, pre- and post-slaughter management, and meat processing and storage methods (Adzitey, 2011; Guerrero et al., 2013; Njisane and Muchenje, 2017). Despite the apparent effectiveness of these alternatives, genetic selection is a complementary approach in which the gains achieved are permanent and cumulative over generations. In this context, carcass and meat quality traits have been measured and incorporated in worldwide beef cattle breeding programs (Reverter et al., 2000; Yokoo et al., 2010; Berry et al., 2017; Gordo et al., 2018). Carcass and meat quality traits can be measured in live animals using ultrasound technology, which is a noninvasive technique (Pathak et al., 2011; Scholz et al., 2015). Ultrasound-based traits that are indicators of carcass and meat quality include *Longissimus* muscle area (LMA), backfat thickness (BFT), rump fat thickness (RFT), and marbling score (MARB) (Pathak et al., 2011; Font-i-Furnols and Guerrero, 2014; Gordo et al., 2018).

Brazil is one of the largest beef cattle producers in the world, with a population of over 230 million animals (USDA, 2019). More than 80% of the beef cattle animals currently raised in Brazil are from the Nellore breed (*Bos taurus indicus*; Zebu), which are well adapted to tropical conditions (Ferraz and Felício, 2010). However, Zebu breeds are also well known for poorer meat quality (Crouse et al., 1989; Bressan et al., 2016; Rodrigues et al., 2017) when compared to Taurine (*Bos taurus taurus*) breeds (*e.g.*, Aberdeen Angus, Red Angus, Senepol, Charolais). An alternative to improve carcass and meat quality traits, while keeping the adaptation characteristics of Zebu cattle, is through the development of composite populations (*i.e.*, crossbreeding between Taurine and Zebu animals; *e.g.* Piccoli et al., 2020).

The Montana Tropical^®^ Composite population was firstly developed in 1994 following studies conducted by the U.S. Meat Animal Research Center at Clay Center, United States Department of Agriculture (USDA; Gregory et al., 1993; Gregory et al., 1994). This composite population was developed by crossing animals from four different biological types or breed groups (Ferraz et al., 1999): 1) Zebu breeds (*Bos taurus indicus*), 2) Adapted Taurine breeds (*Bos taurus taurus*), 3) British breeds (*Bos taurus taurus*), and 4) Continental European breeds (*Bos taurus taurus*).

Over the past few years, there has been a great interest in genetically improving this composite population and better understanding its genetic background underlying phenotypic variation of economic importance to the breeders. In this context, genome-wide association studies (GWAS) can be performed to identify Quantitative Trait Loci (QTL) associated with key traits (*e.g.* carcass and meat quality). Recent GWAS have successfully revealed significant genomic regions in beef cattle composite populations [(*e.g.*, Weng et al., 2016; Hay and Roberts, 2018; Grigoletto et al., 2019)]. Wang et al. (2012) proposed a GWAS method based on the single-step Genomic Best Linear Unbiased Predictor (ssGBLUP; Legarra et al., 2009; Aguilar et al., 2010; Legarra et al., 2014), which has become the gold-standard method for GWAS [also termed single-step Genome-Wide Association Studies (ssGWAS)]. A variation of this method, the weighted single-step GBLUP (WssGBLUP; Wang et al., 2012) usually yields more accurate SNP effects (*e.g.* Zhang et al., 2016), and consequently, a greater power to identify QTLs and functional genes. In this context, the main goals of this study were to: 1) estimate variance components and genetic parameters for four ultrasound-based carcass and meat quality traits (*i.e.*, LMA, BFT, RFT, and MARB) in Montana Tropical^®^ Composite beef cattle and 2) identify relevant genomic regions, candidate genes, and metabolic pathways associated with these traits, using the WssGBLUP method.

## Materials and Methods

Animal Care Committee approval was not obtained for this study as all the analyses were performed using pre-existing databases.

### Animals and Phenotypic Data

**Figure 1:**
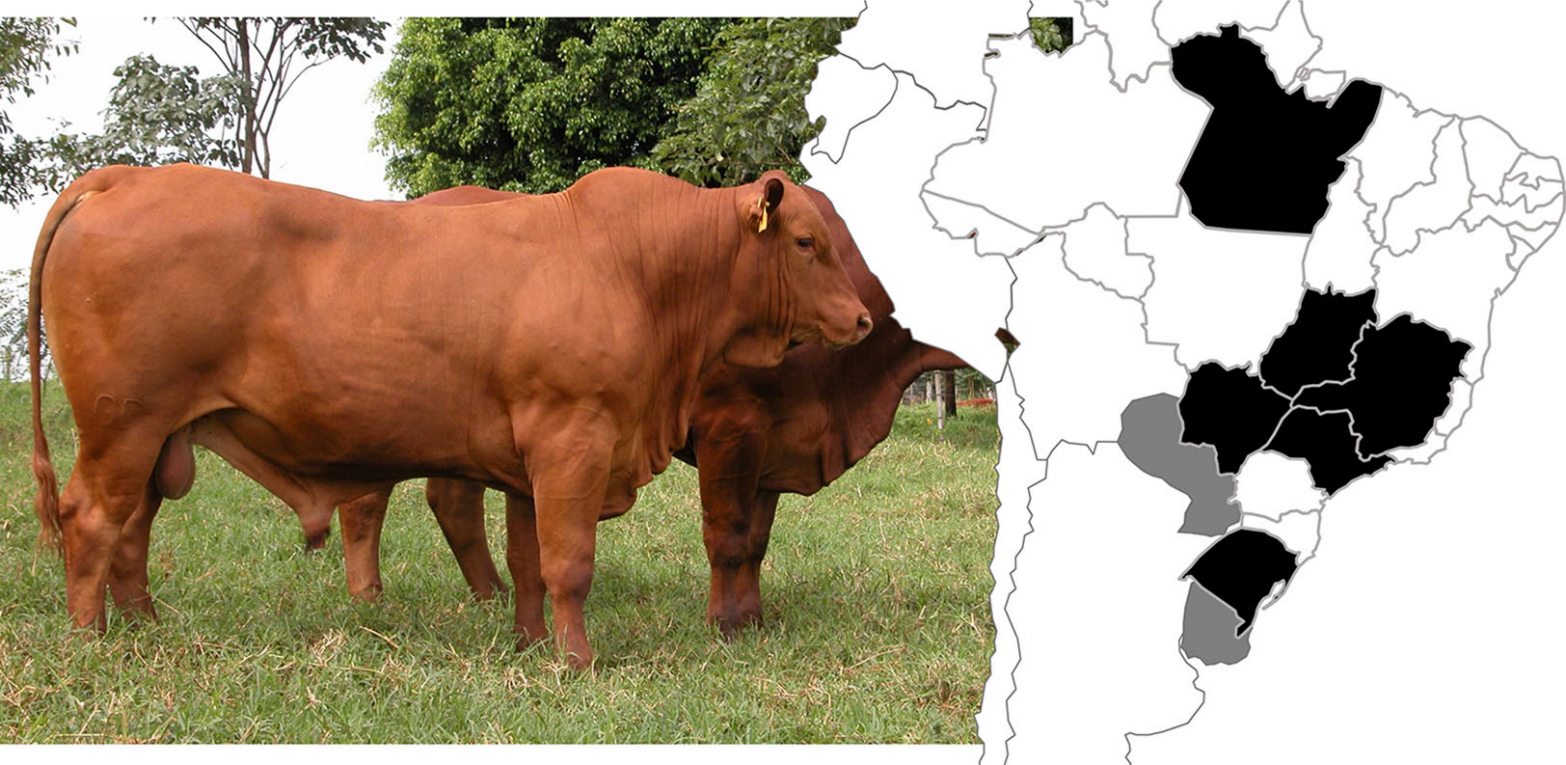
Illustration of a Montana Tropical^®^ Composite bull (left) and location of the farms (right) participating in the Montana Tropical^®^ Composite breeding program. The map regions in black indicate Brazilian states and the gray areas represent Paraguay and Uruguay. Photo Credits: Montana Tropical^®^ Composite website (www.compostomontana.com.br/criadores-montana/).

The descriptive statistics of the pedigree file, including the breed composition of the animals is shown in **Table 1**. Breed was recorded by the producers/technicians or calculated based on pedigree relationship between the animals. The animals were classified within each biological group (NABC) as: 1) N: Zebu breeds, mainly represented by Nellore; 2) A: Taurine breeds adapted to tropical conditions (Senepol, Belmont Red, Bonsmara, and Caracu); 3) B: Taurine breeds of British origin (mainly Angus, Devon, and Hereford); and, 4) C: Continental European breeds (mainly Charolais, Limousin, and Simmental). To be considered as a Montana Tropical^®^ Composite (**Figure 1**), the animals had to have at least three breeds in their genetic composition. In addition, the minimum percentage of the biological types (breed groups) required to be considered a Montana Tropical^®^ Composite was 12.5% for group A and 25% for groups N and A together. The maximum proportion of each group allowed was 37.5% for group N; 87.5% for group A; and 75% for groups B and C (Santana et al., 2013). The main contributing breeds to the development of this composite population were Aberdeen Angus, Red Angus, Nellore, Senepol, Limousin, Simmental, Hereford, and Bonsmara.

**Table 1:** Descriptive statistics of the pedigree dataset according to the breed and biological type composition of the animals.

^1^Biological Type		Number of animals
**Montana Tropical^®^ Composite**	4444	7,136
	4480	4,693
	4804	3,125
	4840	3,127
**Pure breeds**	N ≥ 90%	3,730
	A ≥ 90%	1,461
	B ≥ 90%	1,630
	C ≥ 90%	181
**Crossbreed**	N × A	116
	N × B	2,230
	N × C	842
	A × B	153
	A × C	8
	B × C	48
**Total**		**28,480**

^1^Biological type (NABC system; Ferraz et al., 1999): Zebu breeds (N), Adapted Taurine breeds (A), British Taurine breeds (B), and Continental Taurine breeds (C). Breed composition of the Montana Tropical Composite animals: 4444 = 25% N, A, B and C; 4480 = 25% N, 25% A, 50% B, and < 6.25% C; 4804 = 25% N, 50% A, < 6.25% B, and 25% C; 4840 = 25% N, 50% A, 25% B and < 6.25% C. N × A, N × B, N × C, A × B, A × C, and B × C = the combination of each breed group equals to 50%.

Four ultrasound-based carcass and meat quality traits (LMA, BFT, RFT, and MARB), recorded on animals born between 2008 and 2016, were included in this study. Animals were raised in 18 farms located at different Brazilian states, Paraguay and Uruguay (**Figure 1**). In general, the animals were raised on pastures composed basically of *Brachiaria brizantha*. All farms provided feed supplements in the dry season (from May to August). With regard to the reproductive breeding scheme, around 60% of cows were artificially inseminated and 40% were kept in multiple-sire lots with a cows-to-bull’ ratio of 30:1 or 25:1. The majority of calves were born between September and December (Spring season in South America and the beginning of the rainy period) and weaned at 7 months of age. Weight recording was obtained at birth and weaning. Further records of yearling weight, scrotal circumference, and other productive traits were collected between 14 and 18 months. More details are presented in Santana et al. (2012), and in a previous GWAS study from the same population (Grigoletto et al., 2019).

The average (±standard deviation; SD) age of the animals at the ultrasound measurement was 580.27 (±75.08) days. *Longissimus* muscle area (LMA) was measured in cm^2^, between the 12^th^ and 13^th^ ribs. Backfat thickness (BFT) was measured in mm, at a point three-fourths of transverse orientation over the LMA (Brethour, 2004). Rump fat thickness (RFT) was also measured in mm, at the junction of the *biceps femoris* and *gluteus medius* between the ischium and ilium (Greiner et al., 2003; Gordo et al., 2012). Marbling score (MARB) was measured as an indicator of the percentage of intramuscular fat, using a subjective scale ranging from 1 to 12, based on the U.S. Department of Agriculture (USDA) quality grades (www.uspremiumbeef.com/DocumentItem.aspx?ID=21). All traits were evaluated by ultrasonography using the ALOKA 500 V device, with a 3.5 MHz linear probe. The images were analyzed using the LINCE^®^ software (Gabín et al., 2012). Phenotypic quality control removed records deviating 3.5 SD from the overall mean within contemporary group (CG). The CGs were defined based on farms, years, and seasons of birth, sexes, and management groups. The CGs with less than five records were excluded from subsequent analyses. Descriptive statistics for the ultrasound-based carcass and meat quality traits after the data editing are shown in **Table 2**.

**Table 2:** Descriptive statistics, variance components, and genetic parameter estimate for ultrasound carcass traits in the Montana Tropical^®^ Composite cattle population.

^1^Trait	N	Mean	SD	$\sigma_{a}^{2}$(SE)	$\sigma_{e}^{2}$(SE)	*h*^2^ (SE)
**LMA (cm^2^)**	9,358	58.40	12.79	13.99 (1.73)	33.35 (1.44)	0.29 (0.03)
**BFT (mm)**	5,768	2.84	0.71	0.25 (0.04)	0.68 (0.03)	0.26 (0.03)
**RFT (mm)**	7,996	3.16	1.37	0.09 (0.04)	0.45 (0.03)	0.16 (0.03)
**MARB (score)**	1,972	3.29	1.20	0.18 (0.04)	0.36 (0.04)	0.33 (0.05)

^1^Traits: Longissimus muscle area (LMA); backfat thickness (BFT); rump fat thickness (RFT); marbling score (MARB).

N, number of animals; SD, standard deviation; SE, standard error; $\sigma_{a}^{2}$ *additive genetic variance;* $\sigma_{e}^{2}$ *residual variance; h^2^, heritability*.

### Genotypic Quality Control

A total of 1,436 bulls were genotyped using a moderate-density SNP panel containing 30,105 SNPs (GeneSeek Genomic Profiler™ LDv4-GGP Bovine LDv4; Illumina, San Diego, CA). Genotype quality control was performed using the PREGSF90 program (Aguilar et al., 2014; Aguilar et al., 2019). In general, SNPs with minor allele frequency lower than 0.05, call rate lower than 90%, extreme deviation from Hardy–Weinberg equilibrium (defined as the maximum difference between observed and expected heterozygosity) greater than 0.15 (Wiggans et al., 2009), and SNPs located in nonautosomal chromosomes were excluded. A total of 27,196 SNPs distributed on 29 autosomal chromosomes, and 1,394 genotyped animals (42 animals were excluded due to call rate lower than 90%) remained for further analyses. BTA1 is the largest chromosome, with 158.72 Megabase pairs (Mb) covered by 1,602 SNPs, while the BTA27 is the shortest one, with 42.33 Mb covered by 512 SNPs.

### Statistical Analyses

**Variance components and breeding value prediction**. Single-trait linear animal models and the average-information restricted maximum likelihood (AI-REML) procedure were used to estimate heritability and variance components, using the AIREMLF90 package from the BLUPF90 family programs (Misztal et al., 2002; Misztal et al., 2014). Genomic breeding values for all traits were directly predicted using the ssGBLUP procedure (Misztal et al., 2009; Aguilar et al., 2010; Christensen and Lund, 2010). The ssGBLUP is a modified version of the traditional BLUP, in which the inverse of the pedigree-based relationship matrix (**A**^−1^) is replaced by the **H**^−1^ matrix. The **H**^−1^ is defined as follow (Legarra et al., 2009; Aguilar et al., 2010):

$$H^{-1}=A^{-1}+\left[\begin{array}{ccc} 0 & & 0\\ 0 & & \tau G^{-1}-\omega A_{22}^{-1} \end{array}\right],$$

where **A**^–1^ was previously defined, τ and ω are the scaling factors used to combine **G** and **A_22_** (assumed as τ = 1.0 and ω = 0.7 in order to reduce bias; Misztal et al., 2010; Tsuruta et al., 2011), $A_{22}^{-1}$ is the inverse of the pedigree-based relationship matrix for the genotyped animals, and G^–.^G^-1^ is the inverse of the genomic relationship matrix (**G**), which was calculated as (VanRaden, 2008):

$$G=ZZ^{\prime }/k,$$

where **Z** is the matrix containing the centered genotypes (−1, 0, 1) accounting for the observed allelic frequencies; and k is a scaling parameter, defined as 2 Σ p(1–p), in which p is the observed allele frequency of each marker. The weighting factor can be derived either based on SNP frequencies (VanRaden, 2008) or by ensuring that the average diagonal of **G** is close to one as in **A_22_** (Vitezica et al., 2011). In order to minimize issues with **G** inversion, 0.05 of **A** was added to 0.95 of the **G** matrix.

The single-trait animal models used in this study included the direct additive genetic and residual as random effects. CG, direct (individual) heterozygosity (described below), and age of the animal at the measurement were included as fixed effects in the model. Thus, the statistical model used in this study can be described as:

$$y_{ijkl}=\;CG_{i}+\;b_{1}\left(Age_{j}-\;\bar{Age}\right)+b_{2}\left(H_{D_{k}}-\;\bar{H_{D}}\right)+\;\alpha_{l}+\epsilon_{ijkl},$$

where y_ijkl_ is the phenotypic record for each trait (LMA, BFT, RFT or MARB) recorded on the animal *l*, belonging to the CG *i*, at age *j*, and direct (individual) heterozygosity (H_D_) k. b_1_ and b_2_ are the linear regression coefficients related to the Age and H_D_ effects, respectively, which were considered as deviations from the mean $(\bar{\text{Age}}$ and $\bar{H_{D}})$ The α_1_ is the direct additive genetic random effect for the animal *l*, and ϵ_ijkl_ is the residual random effect associated with the animal *l*, direct (individual) heterozygosity *k*, age *j*, and CG *i*. Assuming a matrix notation, the previous model can be written as:

y=Xβ+Zα+ϵ,

where, **y** is the vector of phenotypic observations for each trait; **β** is the vector of solutions for fixed effects; **α** is the vector of predictions for random additive genetic animal effect; **ϵ** is the vector of random residual terms; **X** and **Z** are the incidence matrices of fixed and random effects, respectively. It was assumed that: α ~ N(0$H\sigma_{\alpha }^{2}$) and ϵ ~ N(0$I\sigma_{\alpha }^{2}$) where $\sigma_{\alpha }^{2}$is the additive genetic variance; $\sigma_{\text{ϵ}}^{2}$is the residual variance; and **I** is an identity matrix. Thus, the (co)variance matrix (**V**) of the random effects can be expressed as:

$$V=\left[\begin{array}{c} H\sigma_{\text{α}}^{2}\\ 0 \end{array}\;\begin{array}{c} 0\\ I\sigma_{\text{ϵ}}^{2} \end{array}\right],$$

where **H** is the relationship matrix used in the ssGBLUP method. The non-additive effects of heterozygosity were obtained by linear regression to the coefficients of direct (individual) heterozygosity (H_D_), which were calculated as (Dias et al., 2011):

$$H_{D}=1-\;\sum_{i=1}^{4}S_{i}D_{i}$$

which *i* represents the biological type (*i.e.*, i = 1, 2, 3 or 4, indicating the proportion of N, A, B, C, respectively); S_i_ and D_i_ are the fractions of the i^th^ biological type of sire and dam, respectively. Coefficients for biological types (N, A, B, and C) were equal to the proportion of each biological type in the breed composition (as recorded by the producers/technicians or calculated based on pedigree relationship between animals), and it was assumed that the sum of all proportions of biological types in one animal were equal to one. To avoid multicollinearity, direct additive effects of the biological type N were excluded from the statistical models, *i.e.*, the effects for A, B, and C were estimated as deviations of the additive effects of N (Dias et al., 2011; Petrini et al., 2012).

**Genetic correlations**. A multiple-trait linear animal model was used to estimate the genetic and phenotypic correlation between all traits (LMA, BFT, RFT, and MARB) using pedigree and genomic information. Genetic and phenotypic correlations were calculated using the AIREMLF90 package from the BLUPF90 family programs (Misztal et al., 2002; Misztal et al., 2014). The multiple-trait model included the same fixed and random effects described above. However, it was assumed that: α ~ N(0, **G⊗H**); ϵ ~ N(0, **R⊗I**); where **α**, **H**, and **I** are the same as above; **G** is the additive genetic (co)variance matrix; **R** is the residual (co)variance matrix. In this reasoning, the (co)variance matrix for random effects was:

$$V=\left[\begin{array}{cc} G⊗H & O\\ 0 & R⊗I \end{array}\right]\;$$

**Genome-wide association studies (GWAS)**. The GWAS for each trait was carried out using the weighted ssGBLUP method (WssGBLUP; Wang et al., 2012). The same statistical models described to estimate the variance components and breeding values were used to identify genomic windows associated with the traits, as described by Wang et al. (2014) using the BLUPF90 family programs (Misztal et al., 2002; Misztal et al., 2014). The PREGSF90 software (Aguilar et al., 2014) was used as an interface to the genomic module to process the genomic information. Also, the POSTGSF90 software (Aguilar et al., 2014) was used to back-solve the GEBVs for each trait. To calculate the SNP effects and weights, we followed the steps proposed by Wang et al. (2014). This method uses an iterative process, which was repeated three times in this study, to increase the weight of SNPs with larger effects and decrease the weight of those markers with smaller (close to zero) effects (Wang et al., 2014). The GWAS results are reported as the proportion of variance explained by a moving genomic window of five adjacent SNPs. Genomic windows that explained more than 1% of the total genetic variance were considered as relevant, *i.e.* associated with the trait being analyzed.

### Functional Analyses

Positional candidate genes were annotated considering an upstream and downstream interval of 100 kb (threshold defined based on the level of linkage disequilibrium in the population) using the Ensembl Genome Browser (www.ensembl.org/index.html) and the ARS-UCD1.2 version of the cattle genome (Zerbino et al., 2017). Furthermore, important SNPs (from the key genomic windows) were further explored using the Animal QTL Database (AnimalQTLdb; Zhi-Liang et al., 2019). Functional analyses were carried out to characterize the gene ontology (GO) terms and Kyoto Encyclopedia of Genes and Genomes (KEGG) pathways using the Database for Annotation, Visualization and Integrated Discovery (DAVID; Huang et al., 2009). In order to increase the statistical power of the study, all candidate genes identified for the four traits were considered in the same functional analysis, as they are all correlated traits. The significance thresholds used were *p*-value < 0.05 and false discovery rate (FDR) < 5 based on the Benjamini-Hochberg procedure (Benjamini and Hochberg, 1995), as implemented in the DAVID software (Huang et al., 2009).

## Results

### Genetic Parameter Estimates

The variance components and heritability (h^2^) estimates for LMA, BFT, RFT, and MARB are presented in **Table 2**. All traits had moderate to high heritability estimates, which ranged from 0.16 ± 0.03 to 0.33 ± 0.05. The genetic and phenotypic correlations are shown in **Table 3**. The highest genetic correlation was obtained between BFT and RFT (0.97 ± 0.02), followed by an unfavorable correlation between BFT and MARB (0.66 ± 0.01). The heritability estimates from the single-trait and averaged bivariate model analyses were similar, and therefore, only the heritability estimates from the single-trait models are reported and discussed here.

**Table 3:** Genetic (above) and phenotypic (below) correlation (±standard error) for ultrasound carcass and meat quality traits in the Montana Tropical Composite beef cattle population.

Traits^1^	LMA	BFT	RFT	MARB
**LMA**		0.46 ± 0.05	0.29 ± 0.08	0.27 ± 0.05
**BFT**	0.53 ± 0.08		0.64 ± 0.03	0.50 ± 0.02
**RFT**	0.39 ± 0.12	0.97 ± 0.02		0.47 ± 0.03
**MARB**	0.23 ± 0.01	0.66 ± 0.01	0.55 ± 0.02	

^1^Traits: Longissimus muscle area (LMA), backfat thickness (BFT), rump fat thickness (RFT) and marbling score (MARB).

### GWAS and Functional Analyses

**Figure 2:**
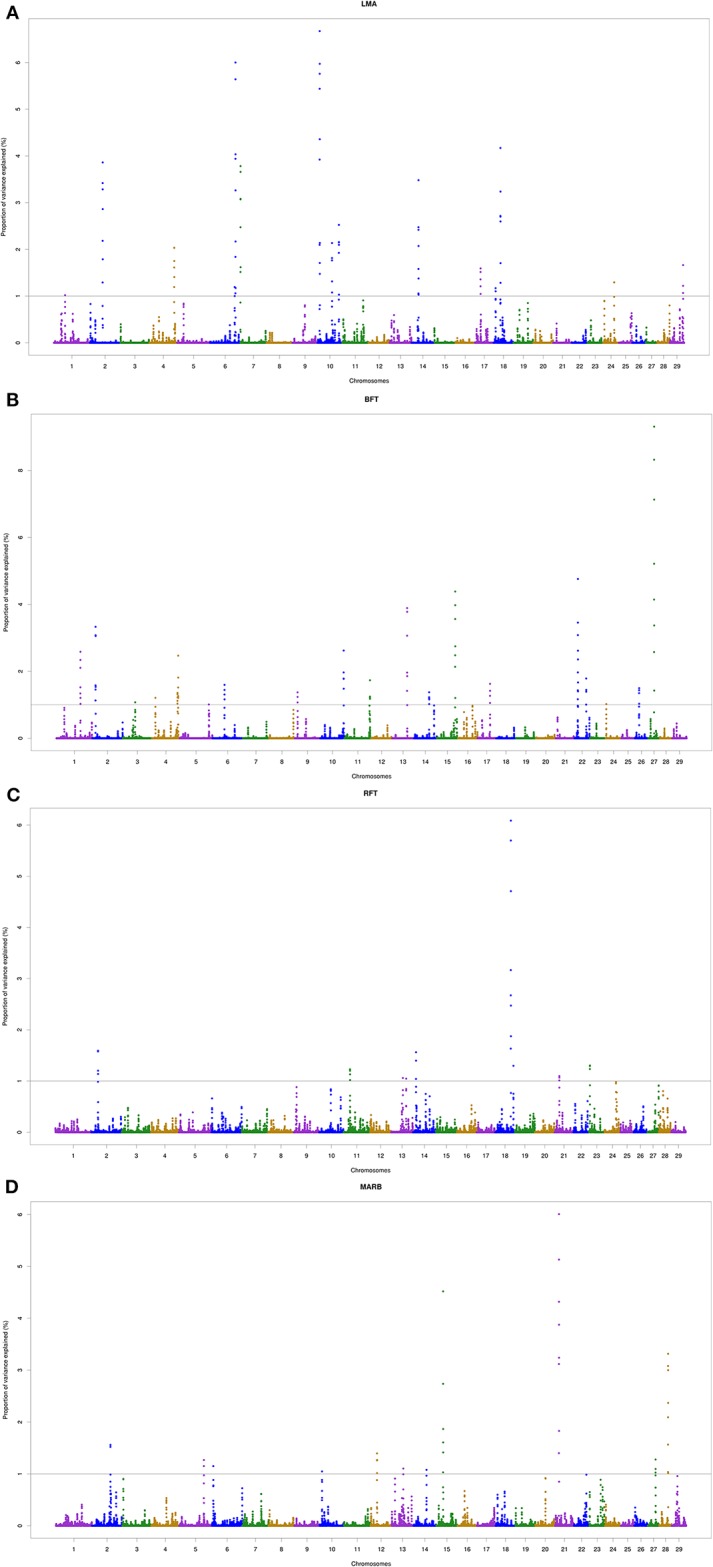
Manhattan plots of the genome-wide association analyses for *Longissimus* muscle area (**A**; LMA), backfat thickness (**B**; BFT), rump fat thickness (**C**; RFT) and marbling score (**D**; MARB) traits. The 29 autosomal chromosomes are shown in different colors. The x-axis represents the chromosome number whereas the y-axis shows the proportion of genetic variance explained by five adjacent SNPs. The gray line corresponds to the genome-wide threshold of each window that explained more than 1% of genetic variance.

A total of 18, 22, 9, and 11 genomic windows explaining more than 1% of the total genetic variance were identified for LMA, BFT, RFT, and MARB, respectively. These regions are harboring or overlap with 241 positional genes. The main candidate genes are shown in **Table 4** and the complete list is presented in the “**Supplementary Material**” section. The genomic windows identified are spread across all autosomal chromosomes, with exception of BTA8, BTA16, BTA19, BTA20, and BTA25 (**Supplementary file** — **Tables S1**–**S4**). The Manhattan plots for all traits are presented in **Figure 2**.

**Table 4:** The main genomic regions explaining more than 1% of total genetic variance (%var) of ultrasound-based carcass traits in the Montana Tropical^®^ Composite beef cattle.

^1^Trait	^2^BTA	Position (start-end, in bp)	%var	Candidate genes
**LMA**	2	64,808,388–65,069,037	3.86	*NCKAP5, LYPD1, GPR39*
	6	102,264,376–102,500,758	6.00	*HSD17B13, HSD17B11, NUDT9, SPARCL1, DSPP, DMP1, PPP2R2C, WFS1, JAKMIP1*
	10	5,392,944–5,807,684	6.67	*HRH2, SFXN1, DRD1*
	14	22,875,603–23,252,097	3.48	*XKR4, TMEM68, TGS1, LYN, RPS20, MOS, PLAG1*
	18	25,832,665–26,209,903	4.17	*KIFC3, CNGB1, TEPP, ZNF319, USB1, MMP15, CFAP20, CSNK2A2, CCDC113, PRSS54, GINS3, NDRG4, SETD6, CNOT1, SLC38A7, GOT2*
**BFT**	2	12,138,830–12,823,369	3.33	*–*
	13	61,566,683–62,224,699	3.88	*PLAGL2, POFUT1, KIF3B, ASXL1, NOL4L, COMMD7, DNMT3B, MAPRE1, EFCAB8, SUN5, BPIFB2*
	15	75,727,954–76,192,434	4.38	*MAPK8IP1, C15H11orf94, PEX16, LARGE2, PHF21A, CREB3L1, CHST1, SLC35C1, CRY2*
	22	12,826,540–13,203,551	4.75	*SCN10A, SCN11A, WDR48, GORASP1, TTC21A, CSRNP1, XIRP1, CX3CR1, CCR8, SLC25A38, RPSA, MOBP, MYRIP, EIF1B, ENTPD3, RPL14, ZNF619, ZNF621, HSPD1*
	27	25,252,766–25,558,906	9.31	*PPP1R3B, TNKS*
**RFT**	2	29,948,707–30,390,796	1.59	*SCN7A, SCN9A, SCN1A, TTC21B*
	14	7,844,432–8,107,746	1.56	*ST3GAL1, NDRG1, CCN4*
	18	60,682,096–61,018,825	6.08	*ZNF331, MGC139164, NLRP12, MGC157082*
	18	65,340,963–65,356,544	1.29	*ZNF814*
	23	3,159,017–3,581,582	1.30	*ZNF451, BEND6*
**MARB**	2	96,082,524–96,725,242	1.55	*PLEKHM3, CRYGD, CRYGC, CRYGB, CRYGA, C2H2orf80, IDH1, PIKFYVE, PTH2R*
	12	22,901,497–23,236,520	1.39	*LHFPL6, NHLRC3, PROSER1, STOML3, FREM2*
	15	24,216,319–24,219,946	4.51	*ZW10*
	21	14,800,548–15,428,801	6.00	*SLCO3A1*
	28	34,157,181–34,514,922	3.31	–

^1^Traits: Longissimus muscle area (LMA), backfat thickness (BFT), rump fat thickness (RFT) and marbling score (MARB). ^2^Bos taurus autosome (BTA).

Two overlapping regions were identified on BTA13: 1) at 47.7–48.5 Mb for BFT and MARB, and 2) at 61.5–63.5 Mb for BFT and RFT. The highest peaks associated with LMA, BFT, RFT, and MARB were located on BTA10 (5.4–5.8 Mb; 6.6% of the genetic variance), BTA27 (25.2–25.5 Mb; 9.3% of the genetic variance), BTA18 (60.6–61.0 Mb; 6.0% of the genetic variance), and BTA21 (14.8–15.4 Mb; 6.0% of the genetic variance), respectively (**Figure 2**). For LMA, a single genomic window was identified on BTA14 (22.8 to 23.2 Mb) explaining close to 4% of the total additive genetic variation. Another region explaining 1.17% of the total additive genetic variance was identified on BTA18 (5.4 to 5.6 Mb) and contains the *WWOX* gene which plays a role in the composition of intramuscular fatty acid associated with cholesterol homeostasis and triglyceride biosynthesis (Iatan et al., 2014). A region located on BTA13 (61.6–62.5 Mb) identified to be associated with both BFT and RFT harbors the candidate genes *PLAGL2*, *ASXL1*, and *BPIFB2*. This suggests that these genes might have pleiotropic effects on BFT and RFT. The genomic region located at BTA22 and harboring the *SCAP* and *ENTPD3* genes accounted for 7.58% of the total genetic variance for BFT. It is worth noting that we highlighted selected genes related to BFT and RFT, however, a total of 13 mutual genes (*HCK, TM9SF4, PLAGL2, POFUT1, KIF3B, ASXL1, NOL4L, COMMD7, DNMT3B, MAPRE1, EFCAB8, SUN5, BPIFB2*) were identified for this common genomic region. A total of 12 biological processes (BP) and two pathways were significantly enriched (**Table 5**). Four biological processes involving visual behavior, associative learning, muscle tissue morphogenesis, and regulation of fatty acid biosynthetic processes were highlighted for further discussion.

**Table 5:** Enriched Gene Ontology (GO) and KEGG terms obtained from the DAVID database (https://david.ncifcrf.gov; Huang et al., 2009).

Category GO	Term	*p*-value	FDR	Genes
**Biological Process**	GO:0008306~associative learning	4.97E-04	0.84	*DDHD2, NDRG4, DRD1, HRH2, LRRN4*
	GO:0007632~visual behavior	6.93E-04	1.17	*DDHD2, NDRG4, DRD1, HRH2, LRRN4*
	GO:0008542~visual learning	0.01	1.53	*DDHD2, NDRG4, DRD1, HRH2, LRRN4*
	GO:0060415~muscle tissue morphogenesis	0.008	2.57	*CCM2L, MYL3, RXRA, ZFPM2*
	GO:0007612~learning	0.014	3.6	*DDHD2, DRD1, LRRN4, HRH2, NDRG4*
	GO:0030817~regulation of cAMP biosynthetic process	0.04	5.2	*DRD1, GALR1, WFS1, GALR3*
	GO:0042304~regulation of fatty acid biosynthetic process	0.01	5.61	*SCAP, INSIG1, PDK4*
	GO:0044060~regulation of endocrine process	0.04	5.4	*FGFR1, CRY2, GALR1*
	GO:0060986~endocrine hormone secretion	0.04	5.9	*FGFR1, CRY2, GALR1*
	GO:0033002~muscle cell proliferation	0.01	7.3	*FGFR1, NDRG4, RXRA, ZFPM2*
	GO:0007611~learning or memory	0.01	8.1	*DDHD2, DRD1, LRRN4, HRH2, NDRG4*
	GO:0001501~skeletal system development	0.02	8.2	*WDR48, FGFR1, CSRNP1, PTH1R, ASXL1, INSIG1, PKDCC, SETD2, WWOX*
**KEGG pathway**	bta00270: Cysteine and methionine metabolism	0.04	14.63	*DNMT3B, GOT2*
	bta04151: PI3K-Akt signaling pathway	0.05	40.64	*CREB3L1, COL5A1, FGFR1, FLT4, PIK3AP1, PPP2R3C, RXRA*

## Discussion

### Genetic Parameters

The genetic parameters obtained for ultrasound carcass traits in the Montana Tropical Composite population (**Table 2**) are similar to literature reports (Mourão et al., 2007). For instance, Meirelles et al. (2010) estimated h^2^ of 0.24 ± 0.09 and 0.33 ± 0.09 for BFT and LMA, respectively, in Canchim beef cattle (a synthetic population based on crossing between Charolais and Zebu breeds). Silva et al. (2019) also reported moderate to high h^2^ estimates for BFT (0.17 ± 0.06), RFT (0.27 ± 0.07), and LMA (0.32 ± 0.02) in Nellore beef cattle. Hay and Roberts (2018) also reported a high h^2^ estimate for LMA (0.32 ± 0.08) in a composite population of 50% Red Angus, 25% Charolais, and 25% Tarentaise beef cattle. The moderate to high heritability estimates indicate that genetic progress can be achieved for these traits through selective breeding.

A high and favorable genetic correlation was estimated between BFT and RFT (*r* = 0.97 ± 0.02), indicating that these traits are controlled by a similar set of genes. Furthermore, this high genetic correlation suggests that indirect genetic progress can be attained by including only one of these two traits in a breeding program. Positive but unfavorable genetic correlations were estimated between BFT and MARB (*r* = 0.66 ± 0.01), RFT and MARB (*r* = 0.55 ± 0.02), LMA and BFT (*r* = 0.53 ± 0.08), and LMA and RFT (*r* = 0.39 ± 0.02). This is because the industry aims to increase MARB and LMA while keeping BFT and RFT at a constant level. However, as these correlations are far from the unit, genetic progress for all the traits can be achieved by including and properly weighting them in a selection index. A favorable correlation was observed between LMA and MARB (*r* = 0.23 ± 0.01). Gordo et al. (2018) also obtained a moderate and positive correlation between LMA and MARB in Zebu cattle. These findings indicate that selection for carcass traits might indirectly improve meat quality.

### GWAS and Functional Analyses

To our best knowledge, this is the first study reporting genomic regions and genetic parameters for carcass and meat quality traits in the Montana Tropical^®^ Composite. The WssGBLUP method enables the inclusion of phenotypes of ungenotyped animals in the GWAS, which improves the accuracy of marker effect estimation (Wang et al., 2012; Aguilar et al., 2019). The genomic regions presented in **Table 4** are harboring candidate genes related to several biological mechanisms associated with carcass and meat quality traits. For instance, the *PPP1R3B* (*protein phosphatase 1, regulatory subunit 3B*) gene was identified to play a role in the expression of all the traits included in this study. *PPP1R3B* has been reported to be associated with meat quality traits in cattle, including pH, meat color, and shear force (Edwards et al., 2003; Kayan, 2011; Cinar et al., 2012), and skeletal muscle development in humans (Munro et al., 2002). In addition, this gene is associated with glucose and glycogen metabolism. Therefore, it may affect the energy availability in skeletal muscle and consequently, contribute to greater muscle growth (Zhao et al., 2019). Also, it regulates deposition of intramuscular fat relative to subcutaneous fat deposition (Choat et al., 2003).

The *PLAGL2*, *CALCR*, *ASXL1*, and *BP1FB*2 genes, identified to be associated with BFT and RFT, play a role in lipid metabolism (Van Dyck et al., 2007). More specifically, *PLAGL2* is part of a subfamily of zinc finger (*PLAG*) gene family proteins (Kas et al., 1998). The *PLAG1* gene, also identified in this study, has a great impact on carcass weight in cattle (Littlejohn et al., 2012). Moreover, many studies have shown that the *PLAG* gene family is a key regulator of mammalian growth and body weight (Littlejohn et al., 2012; Fortes et al., 2013; Utsunomiya et al., 2017; Muramatsu, 2018; Zhang et al., 2019). The *CALCR* gene, located on BTA4 and identified to be associated with BFT, was previously reported to be associated with angularity, body condition score and body depth in Holstein cattle (Magee et al., 2010). The gene *INSIG1* (*Insulin induced gene 1*) has also been associated with growth and carcass traits, including body weight, hip width and withers height (Liu Y. et al., 2012), residual feed intake (Karisa et al., 2013) and milk fatty acids (Rincon et al., 2012). Furthermore, a group of genes (*PLAG1*, *RPS20*, *ATP6V1H*, *RGS20*, *LYN*, *TCEA1*, *MRPL15*, *SOX17*, *RP1, CHCHD7, SDR16C5, SDR16C6, PENK, FAM110B, CYP7A1, SDCBP*) located on a conserved region on BTA14, previously reported as a selective sweep region in dairy and beef cattle breeds (Zhao et al., 2015), might play a crucial role in carcass and meat quality traits. This region seems to be the most relevant association with carcass traits in beef cattle (Magalhães et al., 2016; Hay and Roberts, 2018; Zhang et al., 2019). Furthermore, *LYN, XKR4*, and *TGS1* genes have already been associated with hip height (An et al., 2019), insulin-like growth factor 1 level (Fortes et al., 2012), and carcass traits (including RFT) in Blonde d’Aquitaine, Charolais, Limousine, Belmont Red, Santa Gertrudis, and Nellore cattle (Porto-Neto et al., 2012; Ramayo-Caldas et al., 2014; Magalhães et al., 2016).

The considerable number of common candidate genes (*i.e.*, 114 genes) identified for multiple carcass traits suggests that there are important pleiotropic effects regulating phenotypic expression of these traits. This is also supported by the moderate to high genetic correlation observed here and in other studies (*e.g.* Tonussi et al., 2015; Herd et al., 2018). Recently, Silva et al. (2017) and Hay and Roberts (2018) reported several significant regions on BTA14 associated with BFT and other carcass traits in Zebu and composite beef cattle populations. The genomic region identified on BTA22 (harboring the *SCAP* and *ENTPD3* genes) was also reported by Hay and Roberts (2018) to be associated with BFT in tropical composite cattle. The gene *DNMT3B* (*DNA cytosine-5-methyltransferase 3 beta*), associated with BFT in this study, was previously associated with marbling score, subcutaneous fat, *Longissimus* muscle area, body weight, carcass weight, dressing percentage in offspring of Wagyu and F1 crossbred cows of Limousin with Fuzhou Yellow cattle (Liu X. et al., 2012). *LCORL* has also been previously associated with carcass weight and fat thickness at the 12^th^ rib in crossbred beef cattle (Lindholm-Perry et al., 2011).

The *WWOX* gene, located on BTA18, has been previously associated with meat color in Korean native cattle (Lee et al., 2018). Meat color is one of the main parameters that influence consumers’ preference (Font-i-Furnols and Guerrero, 2014). Additionally, meat color has currently been described to be related to cholesterol homeostasis and fatty acid biosynthesis, which is likely associated with lipid metabolism (Iatan et al., 2014). Furthermore, lipid metabolism in mammals is hypothesized to be associated with immune response and inflammatory processes. This consequently impacts lean deposition and subcutaneous fat deposition, as well as growth rate in cattle (Silva-Vignato et al., 2019).

The number of genotyped animals with phenotypes for the trait(s) of interest and the density of the panel used (number of SNPs after the quality control) are two key factors that influence the identification of important genomic regions, especially those located in regions with low levels of linkage disequilibrium or small effect on the trait. These two factors might have constrained the genomic regions that were identified in this study. However, the SNP panel used in this study contains informative SNPs identified in several breeds, which were also used to develop the Montana Tropical Composite population (Angus, Red Angus, Nellore, Brahman, Charolais, Gelbvieh, Hereford, Limousin, Simmental, Holstein, Jersey, Brown Swiss, Ayrshire, Guernsey, Gyr, Girolando, Brangus, Beefmaster, and Braford). This might have minimized these effects. In view of the limitations described here, further studies using larger datasets and denser SNP panels should be performed to validate the results reported in this study.

### Functional Enrichment Analyses

The moderate to high genetic correlations obtained between RFT and MARB, BFT and RFT, and the common genomic regions and candidate genes identified indicate that muscle development and fat deposition are likely directly correlated processes. Berg and Butterfield (1976) described that as soon as the animal reaches mature age, changes in the proportions of specific tissues are observed. This includes a decrease in muscle-bone growth rates and an increase in fat deposition rate. The two main biological processes identified are: 1) “muscle tissue morphogenesis” (GO:006415) and 2) “regulation of fatty acid biosynthesis” (GO:0042304). A key gene of the muscle tissue morphogenesis is *RXRA* (*Retinoid X receptor, alpha*), which has been associated with weaning weight and yearling weight in Charolais and Brahman cattle (Paredes-Sánchez et al., 2015), and with BFT and meat fatty acids in an Angus–Hereford–Limousin crossbred population (Goszczynski et al., 2016). The fatty acid composition is directly linked with intramuscular fat content, and its major regulation is located in the skeletal muscle in mammals (Muoio et al., 2002). Meat fatty acid content is a crucial parameter of consumers acceptability and might become a key breeding goal in Nellore cattle (*e.g.*, Lemos et al., 2016; Feitosa et al., 2017; Feitosa et al., 2019), one of the most influential breeds in the development of the Montana Composite population. In general, meat fatty acid content is related to meat quality and flavor and complex interactions occurring during the animals’ life and *post-mortem* period (Mullen et al., 2006).

Two of the highlighted processes are related to behavior indicator traits: 1) visual behavior and 2) associative learning. The associative learning is defined as the capacity of an individual learning a behavior based on the association of two or more events (Abramson and Kieson, 2016). In general, animals recognize events related to environmental factors through this process. For example, the animal's temperament from previous handling experiences produces an active learning process to determine how it will react in a next handling event. Furthermore, mounting behavior can result in carcass bruising and thus reduce carcass quality especially depending on the level of BFT (Hoffman and Lühl, 2012). This is a very important finding, as cattle temperament is significantly associated with handling stress and consequently, carcass damage, and reduction in meat quality (Yang et al., 2019). The association between visual behavior and associative learning processes can also be related to feeding behavior which is a relevant process associated with feed efficiency, growth rate, and carcass composition.

The KEGG pathway PI3K-AKT is associated with stimulation of cell growth and proliferation, and simultaneously inhibits apoptosis. In this regard, PI3Ks plays a major role in insulin metabolism (Ma et al., 2017), which is the major hormone controlling glucose and lipid metabolism (Dimitriadis et al., 2011). In this context, Shingu et al. (2001) suggested that insulin secretion may contribute to the difference in growth patterns and meat quality properties among beef cattle breeds. Another pathway enriched was “bta00270: Cysteine and methionine metabolism”, which is associated with meat flavor development in several species (Mecchi et al., 1964; Minor et al., 1965; Pepper and Pearson, 1969; Pippen et al., 1969), and likely associated with intramuscular fat (or MARB). Cysteine and methionine are considered the largest components of meat flavor (Werkhoff et al., 1990; Khan et al., 2015). Uncooked meat has little to no aroma and only a blood-like taste, thus, the meat flavor is thermally derived by reactions between carbohydrates and amino acids (Mottram, 1998).

## Conclusions

Our findings indicate that ultrasound-based carcass and meat quality traits are heritable and therefore can be improved through selective breeding. The high genetic correlation between BFT and RFT indicate that indirect genetic response can be obtained by selecting for only one of them. The WssGBLUP method used to perform GWAS enabled the identification of various novel or already known candidate genes associated with the carcass and meat quality traits in the Montana Tropical^®^ Composite population, but the traits studied have a polygenic nature. Some of the genes identified were previously associated with traits such as growth, carcass, body condition score, skeletal muscle growth, carcass fatness, and meat fatty acid composition. The main biological processes and pathways identified were “muscle tissue morphogenesis” and “regulation of fatty acid biosynthetic”, which biologically validate the ultrasound-based measurements. Further studies using larger datasets (ideally in independent populations) and denser SNP panels (>30 K) should be performed in order to validate the results reported in this study.

## Data Availability Statement

The data supporting the results of this article are included within the article/**Supplementary Material**. The raw data cannot be made publicly available, as it is property of the Montana Tropical Composite breeders and this information is commercially sensitive. Reasonable requests for access to the raw datasets for research purposes can be e-mailed to: jbferraz@usp.br (JF).

## Author Contributions

LG, JF, FB, and LB conceived and designed the project. LG, JF, JE, FOB, BS organized sample collection and genotyping. LG performed the analyses. LG, LB, and HO wrote the manuscript. All authors reviewed and approved the final version of the manuscript.

## Funding

This study was financially supported by the Sao Paulo Research Foundation (Fundação de Amparo à Pesquisa do Estado de São Paulo — FAPESP) through the grants: 2014/07566-2, 2017/11919-6, and 2018/20393-0.

## Conflict of Interest

The authors declare that the research was conducted in the absence of any commercial or financial relationships that could be construed as a potential conflict of interest.

The reviewer AM declared a shared affiliation, with no collaboration, with one of the authors, FB, to the handling editor at time of review.
