# Supplementary material for: Genetic Architecture of Carcass and Meat Quality Traits in Montana Tropical® Composite Beef Cattle
Source: Front Genet. 2020 Feb 27;11:123. doi: 10.3389/fgene.2020.00123 (PMC7057717; doi:10.3389/fgene.2020.00123)
Supplement: Supplementary file 1 [file DataSheet_1.docx]

Supplementary Material

**Table S1.** Genome-wide regions and candidate genes that explain more than 1% of the total genetic variance for *Longissimus* muscle area (LMA) in the Montana Tropical Composite beef cattle population.

| BTA | Position (bp) | | Gene name |
| --- | --- | --- | --- |
|  | **Start** | **End** |  |
| 1 | 51,940,576 | 51,941,806 | *CCDC54* |
| 2 | 64,157,472 | 64,872,456 | *NCKAP5* |
| 2 | 64,874,976 | 64,937,074 | *LYPD1* |
| 2 | 64,935,007 | 65,196,499 | *GPR39* |
| 4 | 12,191,129 | 12,534,305 | *PPP1R9A* |
| 4 | 12,542,354 | 12,576,328 | *PON1* |
| 4 | 12,593,909 | 12,631,421 | *PON3* |
| 4 | 12,645,430 | 12,673,193 | *PON2* |
| 4 | 12,750,329 | 12,843,879 | *ASB4* |
| 4 | 12,881,889 | 12,895,362 | *PDK4* |
| 4 | 13,061,840 | 13,440,805 | *DYNC1I1* |
| 6 | 102,192,504 | 102,212,283 | *HSD17B13* |
| 6 | 102,227,496 | 102,276,940 | *HSD17B11* |
| 6 | 102,320,394 | 102,357,766 | *NUDT9* |
| 6 | 102,370,630 | 102,423,199 | *SPARCL1* |
| 6 | 102,494,936 | 102,500,867 | *DSPP* |
| 6 | 102,526,308 | 102,543,087 | *DMP1* |
| 6 | 102,716,789 | 102,881,084 | *PPP2R2C* |
| 6 | 102,896,904 | 102,928,920 | *WFS1* |
| 6 | 103,013,174 | 103,169,423 | *JAKMIP1* |
| 7 | 496,572 | 537,888 | *FLT4* |
| 10 | 5,450,044 | 5,474,341 | *HRH2* |
| 10 | 5,613,866 | 5,674,977 | *SFXN1* |
| 10 | 5,715,882 | 5,718,113 | *DRD1* |
| 14 | 22,640,221 | 22,953,771 | *XKR4* |
| 14 | 23,034,280 | 23,070,124 | *TMEM68* |
| 14 | 23,070,145 | 23,095,949 | *TGS1* |
| 14 | 23,134,995 | 23,244,752 | *LYN* |
| 14 | 23,278,316 | 23,279,689 | *RPS20* |
| 14 | 23,299,177 | 23,300,199 | *MOS* |
| 14 | 23,330,541 | 23,375,751 | *PLAG1* |
| 18 | 25,722,016 | 25,756,803 | *KIFC3* |
| 18 | 25,813,885 | 25,882,542 | *CNGB1* |
| 18 | 25,888,368 | 25,895,551 | *TEPP* |
| 18 | 25,902,577 | 25,904,786 | *ZNF319* |
| 18 | 25,907,856 | 25,923,157 | *USB1* |
| 18 | 25,939,947 | 25,960,720 | *MMP15* |
| 18 | 25,994,990 | 26,008,531 | *CFAP20* |
| 18 | 26,016,093 | 26,051,043 | *CSNK2A2* |
| 18 | 26,085,843 | 26,119,863 | *CCDC113* |
| 18 | 26,119,132 | 26,132,035 | *PRSS54* |
| 18 | 26,198,116 | 26,204,583 | *GINS3* |
| 18 | 26,255,289 | 26,298,424 | *NDRG4* |
| 18 | 26,299,649 | 26,305,367 | *SETD6* |
| 18 | 26,304,976 | 26,388,347 | *CNOT1* |
| 18 | 26,429,234 | 26,440,641 | *SLC38A7* |
| 18 | 26,447,740 | 26,471,387 | *GOT2* |
| 18 | 5,240,349 | 6,170,333 | *WWOX* |
| 24 | 47,693,446 | 47,994,499 | *ZBTB7C* |
| 24 | 48,220,180 | 48,542,521 | *CTIF* |
| 29 | 49,970,526 | 49,980,774 | *DUSP8* |
| 29 | 49,990,564 | 50,043,414 | *MOB2* |

BTA: *Bos taurus* Autossome.

**Table S2.** Genome-wide regions and candidate genes that explain more than 1% of the total genetic variance for backfat thickness (BFT) in the Montana Tropical Composite beef cattle population.

| BTA | Position (bp) | | Gene name |
| --- | --- | --- | --- |
|  | **Start** | **End** |  |
| 1 | 114,908,447 | 114,910,097 | *P2RY1* |
| 1 | 115,303,718 | 115,524,968 | *MBNL1* |
| 4 | 10,600,800 | 10,732,873 | *VPS50* |
| 4 | 10,781,954 | 10,893,236 | *CALCR* |
| 4 | 117,125,786 | 117,138,560 | *INSIG1* |
| 4 | 117,222,455 | 117,226,427 | *EN2* |
| 4 | 117,261,920 | 117,301,793 | *CNPY1* |
| 4 | 117,366,283 | 117,462,802 | *RBM33* |
| 5 | 109,423,829 | 109,432,050 | *CDC42EP1* |
| 5 | 109,432,450 | 109,438,746 | *LGALS2* |
| 5 | 109,458,483 | 109,477,483 | *GGA1* |
| 5 | 109,484,519 | 109,497,008 | *SH3BP1* |
| 5 | 109,498,401 | 109,504,537 | *PDXP* |
| 5 | 109,512,489 | 109,515,793 | *LGALS1* |
| 5 | 109,519,065 | 109,526,829 | *NOL12* |
| 5 | 109,532,805 | 109,591,861 | *TRIOBP* |
| 5 | 109,621,889 | 109,634,246 | *GCAT* |
| 5 | 109,630,804 | 109,639,001 | *GALR3* |
| 5 | 109,643,325 | 109,653,939 | *ANKRD54* |
| 5 | 109,662,810 | 109,683,993 | *EIF3L* |
| 5 | 109,709,317 | 109,737,117 | *MICALL1* |
| 5 | 109,738,012 | 109,745,876 | *C5H22orf23* |
| 5 | 109,745,905 | 109,755,102 | *POLR2F* |
| 5 | 109,757,715 | 109,768,623 | *SOX10* |
| 5 | 109,834,675 | 109,851,541 | *PICK1* |
| 5 | 109,853,974 | 109,858,626 | *SLC16A8* |
| 5 | 109,861,367 | 109,891,295 | *BAIAP2L2* |
| 5 | 109,891,806 | 109,938,952 | *PLA2G6* |
| 9 | 10,397,908 | 10,413,050 | *OGFRL1* |
| 10 | 101,466,634 | 101,634,822 | *EFCAB11* |
| 10 | 101,640,018 | 101,708,726 | *TDP1* |
| 10 | 101,723,038 | 101,828,432 | *KCNK13* |
| 11 | 105,021,683 | 105,114,871 | *RXRA* |
| 11 | 105,268,089 | 105,419,303 | *COL5A1* |
| 11 | 105,440,661 | 105,450,692 | *FCN1* |
| 13 | 61,464,644 | 61,471,874 | *PDRG1* |
| 13 | 61,487,256 | 61,512,327 | *XKR7* |
| 13 | 61,525,479 | 61,545,528 | *CCM2L* |
| 13 | 61,563,070 | 61,608,503 | *HCK* |
| 13 | 61,622,078 | 61,675,062 | *TM9SF4* |
| 13 | 61,683,029 | 61,694,301 | *PLAGL2* |
| 13 | 61,694,343 | 61,745,905 | *POFUT1* |
| 13 | 61,747,316 | 61,787,770 | *KIF3B* |
| 13 | 61,807,148 | 61,871,197 | *ASXL1* |
| 13 | 61,874,277 | 61,955,381 | *NOL4L* |
| 13 | 62,102,412 | 62,131,186 | *COMMD7* |
| 13 | 62,142,537 | 62,176,363 | *DNMT3B* |
| 13 | 62,182,219 | 62,210,102 | *MAPRE1* |
| 13 | 62,220,966 | 62,268,229 | *EFCAB8* |
| 13 | 62,276,784 | 62,297,291 | *SUN5* |
| 13 | 62,304,795 | 62,323,150 | *BPIFB2* |
| 14 | 58,819,937 | 59,239,114 | *ZFPM2* |
| 15 | 75,918,764 | 75,938,190 | *MAPK8IP1* |
| 15 | 75,938,260 | 75,938,905 | *C15H11orf94* |
| 15 | 75,941,284 | 75,946,922 | *PEX16* |
| 15 | 75,950,774 | 75,956,775 | *LARGE2* |
| 15 | 75,955,555 | 76,148,892 | *PHF21A* |
| 15 | 76,282,608 | 76,319,986 | *CREB3L1* |
| 15 | 75,702,165 | 75,705,849 | *CHST1* |
| 15 | 75,852,557 | 75,860,286 | *SLC35C1* |
| 15 | 75,879,778 | 75,915,413 | *CRY2* |
| 17 | 55,569,766 | 55,740,689 | *CIT* |
| 17 | 55,750,475 | 55,759,908 | *PRKAB1* |
| 17 | 55,795,742 | 55,830,506 | *TMEM233* |
| 17 | 55,876,002 | 56,046,137 | *CCDC60* |
| 17 | 56,156,718 | 56,169,968 | *HSPB8* |
| 17 | 56,189,388 | 56,355,937 | *SRRM4* |
| 22 | 12,106,802 | 12,195,313 | *SCN10A* |
| 22 | 12,236,370 | 12,317,469 | *SCN11A* |
| 22 | 12,377,233 | 12,429,953 | *WDR48* |
| 22 | 12,431,752 | 12,443,560 | *GORASP1* |
| 22 | 12,455,223 | 12,477,183 | *TTC21A* |
| 22 | 12,478,903 | 12,492,412 | *CSRNP1* |
| 22 | 12,509,227 | 12,518,446 | *XIRP1* |
| 22 | 12,569,452 | 12,583,576 | *CX3CR1* |
| 22 | 12,634,614 | 12,635,672 | *CCR8* |
| 22 | 12,645,751 | 12,660,388 | *SLC25A38* |
| 22 | 12,684,159 | 12,710,177 | *RPSA* |
| 22 | 12,723,390 | 12,756,307 | *MOBP* |
| 22 | 12,922,611 | 13,149,096 | *MYRIP* |
| 22 | 13,170,136 | 13,172,696 | *EIF1B* |
| 22 | 13,227,438 | 13,261,914 | *ENTPD3* |
| 22 | 13,292,655 | 13,296,110 | *RPL14* |
| 22 | 13,302,897 | 13,310,529 | *ZNF619* |
| 22 | 13,325,988 | 13,336,394 | *ZNF621* |
| 22 | 13,635,727 | 13,637,448 | *HSPD1* |
| 22 | 52,089,753 | 52,102,048 | *CSPG5* |
| 22 | 52,133,185 | 52,149,780 | *ELP6* |
| 22 | 52,163,455 | 52,264,116 | *SCAP* |
| 22 | 52,263,501 | 52,284,441 | *PTPN23* |
| 22 | 52,297,736 | 52,301,963 | *NGP* |
| 22 | 52,310,680 | 52,361,884 | *KLHL18* |
| 22 | 52,361,996 | 52,398,789 | *KIF9* |
| 22 | 52,450,147 | 52,509,045 | *SETD2* |
| 22 | 52,511,365 | 52,515,665 | *NRADD* |
| 22 | 52,548,484 | 52,585,071 | *CCDC12* |
| 22 | 52,597,375 | 52,624,332 | *PTH1R* |
| 22 | 52,638,016 | 52,644,842 | *MYL3* |
| 22 | 52,671,297 | 52,675,596 | *PRSS42P* |
| 22 | 52,731,581 | 52,739,251 | *PRSS45* |
| 24 | 2,145,784 | 2,156,850 | *GALR1* |
| 24 | 2,224,463 | 2,328,579 | *MBP* |
| 24 | 2,336,527 | 2,399,019 | *ZNF236* |
| 26 | 17,925,643 | 18,029,878 | *PIK3AP1* |
| 26 | 18,219,077 | 18,256,221 | *LCOR* |
| 26 | 18,272,204 | 18,444,938 | *SLIT1* |
| 27 | 25,180,847 | 25,190,311 | *PPP1R3B* |
| 27 | 25,497,637 | 25,654,276 | *TNKS* |

BTA: *Bos taurus* Autossome.

**Table S3.** Genome-wide regions and candidate genes that explain more than 1% of the total genetic variance for rump fat thickness (RFT) in the Montana Tropical Composite beef cattle population.

| BTA | Position (bp) | | Gene name |
| --- | --- | --- | --- |
|  | **Start** | **End** |  |
| 2 | 29,840,643 | 29,898,509 | *SCN7A* |
| 2 | 29,996,676 | 30,088,178 | *SCN9A* |
| 2 | 30,224,956 | 30,320,883 | *SCN1A* |
| 2 | 30,336,543 | 30,434,725 | *TTC21B* |
| 11 | 24,464,835 | 24,474,936 | *PKDCC* |
| 11 | 24,585,989 | 24,739,169 | *EML4* |
| 11 | 24,756,935 | 24,769,582 | *COX7A2L* |
| 11 | 24,832,853 | 24,885,462 | *KCNG3* |
| 11 | 24,886,922 | 25,096,963 | *MTA3* |
| 13 | 47,916,971 | 48,028,742 | *SHLD1* |
| 13 | 48,069,187 | 48,084,157 | *CHGB* |
| 13 | 48,110,221 | 48,120,755 | *TRMT6* |
| 13 | 48,123,958 | 48,166,976 | *MCM8* |
| 13 | 48,186,875 | 48,209,791 | *CRLS1* |
| 13 | 48,213,047 | 48,233,309 | *LRRN4* |
| 13 | 48,251,375 | 48,312,017 | *FERMT1* |
| 13 | 61,622,078 | 61,675,062 | *TM9SF4* |
| 13 | 61,683,029 | 61,694,301 | *PLAGL2* |
| 13 | 61,694,343 | 61,745,905 | *POFUT1* |
| 13 | 61,747,316 | 61,787,770 | *KIF3B* |
| 13 | 61,807,148 | 61,871,197 | *ASXL1* |
| 13 | 61,874,277 | 61,955,381 | *NOL4L* |
| 13 | 62,102,412 | 62,131,186 | *COMMD7* |
| 13 | 62,142,537 | 62,176,363 | *DNMT3B* |
| 13 | 62,182,219 | 62,210,102 | *MAPRE1* |
| 13 | 62,220,966 | 62,268,229 | *EFCAB8* |
| 13 | 62,276,784 | 62,297,291 | *SUN5* |
| 13 | 62,304,795 | 62,323,150 | *BPIFB2* |
| 13 | 62,331,559 | 62,347,090 | *BPIFB6* |
| 13 | 62,356,043 | 62,372,303 | *BPIFB3* |
| 13 | 62,381,695 | 62,408,299 | *BPIFB4* |
| 14 | 7,865,552 | 7,951,420 | *ST3GAL1* |
| 14 | 8,073,208 | 8,129,604 | *NDRG1* |
| 14 | 8,137,189 | 8,169,248 | *CCN4* |
| 18 | 60,741,854 | 60,749,312 | *ZNF331* |
| 18 | 60,856,348 | 60,866,029 | *MGC139164* |
| 18 | 60,905,906 | 60,925,131 | *NLRP12* |
| 18 | 61,009,582 | 61,018,636 | *MGC157082* |
| 18 | 65,375,097 | 65,405,941 | *ZNF814* |
| 21 | 18,837,293 | 19,260,319 | *NTRK3* |
| 21 | 19,474,861 | 19,484,599 | *MRPL46* |
| 21 | 19,484,255 | 19,495,138 | *MRPS11* |
| 21 | 19,522,497 | 19,547,431 | *DET1* |
| 21 | 19,591,620 | 19,601,252 | *AEN* |
| 21 | 19,607,426 | 19,625,071 | *ISG20* |
| 23 | 3,051,079 | 3,142,445 | *ZNF451* |
| 23 | 3,189,366 | 3,252,666 | *BEND6* |

BTA: *Bos taurus* Autossome.

**Table S4.** Genome-wide regions and candidate genes that explain more than 1% of the total genetic variance for marbling score (MARB) in the Montana Tropical Composite beef cattle population.

| BTA | Position (bp) | | Gene name |
| --- | --- | --- | --- |
|  | **Start** | **End** |  |
| 2 | 96,101,252 | 96,318,495 | *PLEKHM3* |
| 2 | 96,395,739 | 96,397,590 | *CRYGD* |
| 2 | 96,405,966 | 96,407,968 | *CRYGC* |
| 2 | 96,417,867 | 96,420,083 | *CRYGB* |
| 2 | 96,432,628 | 96,435,050 | *CRYGA* |
| 2 | 96,437,069 | 96,460,618 | *C2H2orf80* |
| 2 | 96,510,261 | 96,532,002 | *IDH1* |
| 2 | 96,557,844 | 96,625,577 | *PIKFYVE* |
| 2 | 96,667,717 | 96,752,328 | *PTH2R* |
| 5 | 94,669,118 | 94,824,113 | *RERG* |
| 6 | 4,138,065 | 4,200,076 | *QRFPR* |
| 10 | 7,084,931 | 7,115,694 | *POC5* |
| 10 | 7,361,671 | 7,574,347 | *SV2C* |
| 12 | 22,798,940 | 23,004,081 | *LHFPL6* |
| 12 | 23,178,045 | 23,219,416 | *NHLRC3* |
| 12 | 23,219,451 | 23,251,116 | *PROSER1* |
| 12 | 23,258,105 | 23,284,263 | *STOML3* |
| 12 | 23,297,835 | 23,449,177 | *FREM2* |
| 13 | 47,720,768 | 47,778,950 | *GPCPD1* |
| 13 | 47,916,971 | 48,028,742 | *SHLD1* |
| 13 | 48,069,187 | 48,084,157 | *CHGB* |
| 13 | 48,110,221 | 48,120,755 | *TRMT6* |
| 13 | 48,123,958 | 48,166,976 | *MCM8* |
| 13 | 48,186,875 | 48,209,791 | *CRLS1* |
| 13 | 48,213,047 | 48,233,309 | *LRRN4* |
| 13 | 48,251,375 | 48,312,017 | *FERMT1* |
| 14 | 48,629,593 | 48,904,459 | *TRPS1* |
| 15 | 24,284,251 | 24,319,569 | *ZW10* |
| 21 | 14,842,445 | 15,219,453 | *SLCO3A1* |
| 27 | 33,387,283 | 33,416,172 | *DDHD2* |
| 27 | 33,416,459 | 33,421,456 | *PLPP5* |
| 27 | 33,427,742 | 33,524,431 | *NSD3* |
| 27 | 33,528,788 | 33,544,151 | *LETM2* |
| 27 | 33,549,267 | 33,599,083 | *FGFR1* |

BTA: *Bos taurus* Autossome.
